# Self‐harm and suicidal ideation in children and adolescents in contact with child protection services

**DOI:** 10.5694/mja2.51898

**Published:** 2023-03-27

**Authors:** Kirstie O'Hare, Oliver Watkeys, Felicity Harris, Kimberlie Dean, Vaughan J Carr, Melissa J Green

**Affiliations:** ^1^ The University of New South Wales Sydney NSW; ^2^ Justice Health and Forensic Mental Health Network Sydney NSW; ^3^ Neuroscience Research Australia Sydney NSW; ^4^ Monash University Melbourne VIC

**Keywords:** Suicide, Child psychiatry, Child protective services, Self‐injurious behavior, Longitudinal studies

Self‐harm and suicide by young people are major problems,^1^ and they are more frequent among people known to child protection services,^2^, ^3^ especially those placed in out‐of‐home care.^4^ We used record linkage to quantify the cumulative incidence of self‐harm and suicidal ideation in a population sample of New South Wales children and adolescents aged 0–17 years with differing levels of child protection contact during 2003–21.

We analysed data for the 91 597 participants in wave 3 of the New South Wales Child Development Study^5^ (http://nsw‐cds.com.au). First incidents of self‐harm (International Classification of Diseases, tenth revision, Australian modification [ICD‐10‐AM] codes X60–X84, T14.91, Y87.0) or suicidal ideation (ICD‐10‐AM codes R45.81, R45.851) during 1 January 2000 – 31 March 2021 were identified in linked health records in the NSW and Australian Capital Territory emergency department, admitted patient, and mental health ambulatory data collections. Record linkage was conducted by the Centre for Health Record Linkage (http://www.cherel.org.au), with an estimated false positive linkage rate of 0.5%. Young people with records of both self‐harm and suicidal ideation were included in the self‐harm group.

Child protection contact was identified in the NSW Department of Communities and Justice ChildStory database^6^ (1 July 1998 – 31 July 2021). Each participant was assigned to one of four categories of child protection contact according to the highest level: out‐of‐home care, substantiated report of child abuse or neglect non‐substantiated/non‐threshold report of child abuse or neglect, and no child protection contact. The cumulative incidence of first incidents of self‐harm and suicidal ideation was calculated for each child protection level, based on the cumulative number of people at each year of age in the study sample (further details: Supporting Information). The NSW Population and Health Services, ACT Health, and Calvary Public Hospital human research ethics committees approved the study (HREC/18/CIPHS/49).

By age 17 years, incidents of self‐harm or suicidal ideation were recorded in medical records for 2233 study participants (2.4%) (Box 1); 866 incidents involved self‐harm, 1367 involved suicidal ideation (without self‐harm). By age 17, incidents had been recorded for 1505 girls (incidence, 3.4%) and 728 boys (incidence, 1.5%). Contact with child protection services was recorded for 1671 young people with records of self‐harm or suicidal ideation incidents (74.8%). The cumulative incidence of self‐harm or suicidal ideation by 17 years was 13.0% for young people with out‐of‐home care placements, 10.4% for those with substantiated reports, 4.6% for those with non‐substantiated/non‐threshold reports, and 0.9% for those who had not come into contact with child protection services (Box 2). A similar pattern pertained to separate analyses of self‐harm and suicidal ideation (Box 2), and to separate analyses by gender (Supporting Information, figure).

Box 1 **Characteristics of the New South Wales Child Development Study (wave 3) participants**

**Table:** 

Characteristic	Value
Number of people aged 0–17 years	91 597
Gender (boys)	47 381 (51.7%)
Aboriginal or Torres Strait Islander	7970 (8.7%)
Child protection contact (highest level)	
None	64 851 (70.8%)
Non‐substantiated/non‐threshold report of abuse/neglect	20 083 (21.9%)
Substantiated report of abuse/neglect	4325 (4.7%)
Out‐of‐home care	2338 (2.6%)
Recorded incidents of self‐harm or suicidal ideation (by 17 years of age)	2233 (2.4%)
Any self‐harm	866 (0.9%)
Any suicidal ideation*	1367 (1.5%)

* Excludes people with recorded incidents of self‐harm.

Box 2 **Cumulative relative incidence of first incidents of self‐harm and suicidal ideation recorded in health care records of the 91 597 participants in the New South Wales Child Development Study (wave 3)**

**Figure:**
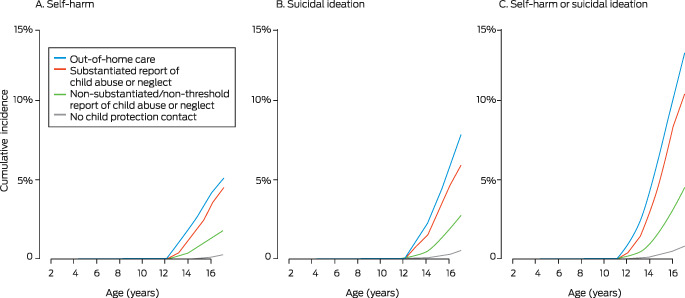
* The data on which these graphs are based are provided in the Supporting Information, table 1.

Not all incidents of self‐harm or suicidal ideation will have been captured in administrative health records.^7^ Our study is descriptive and no causal inferences should be drawn with regard to the impact of child protection service contact on the likelihood of self‐harm or suicidal ideation. We could not determine whether contact with child protection services preceded individual incidents, but 21 223 child protection contact episodes took place before the age of twelve years (79.3%), while 2139 first self‐harm and suicidal ideation incidents (95.7%) happened after age eleven years.

As almost three‐quarters of recorded self‐harm and suicidal ideation incidents requiring health care support involved young people who had experienced contact with child protection services, targeted prevention and intervention strategies are needed. Children and adolescents who have had contact with child protection services may benefit from screening for mental health problems, particularly for risks of self‐harm and suicidal ideation. The high incidence of self‐harm and suicidal ideation among young people who have contact with child protection services confirms that child maltreatment is an important public health concern.

## Open access

Open access publishing facilitated by University of New South Wales, as part of the Wiley ‐ University of New South Wales agreement via the Council of Australian University Librarians.

## Competing interests

No relevant disclosures.

## Supporting information


Supplementary methods and results.
