# Supplementary material for: Self‐harm and suicidal ideation in children and adolescents in contact with child protection services
Source: Med J Aust. 2023 Mar 27;218(11):526–7. doi: 10.5694/mja2.51898 (PMC10952315; doi:10.5694/mja2.51898)
Supplement: Supplementary file 1 — Supplementary methods and results. [file MJA2-218-526-s001.pdf]

The Medical Journal of Australia

Supporting Information

Supplementary methods and results

This appendix was part of the submitted manuscript and has been peer reviewed. It is posted as supplied by the authors.

Appendix to: O'Hare K, Watkeys O, Harris F, et al. Self-harm and suicidal ideation in children and adolescents in contact with child protection services. *Med J Aust* 2023; doi: 10.5694/mja2.51898.

Supplementary methods

Individuals were classified into mutually exclusive groups based on their highest level of suicide risk, regardless of which incident had occurred first: (1) self-harm, followed by (2) suicidal ideation. Individuals were also classified into four mutually exclusive groups representing their highest level of child protection service provision: (1) out-of-home care, (2) substantiated report, (3) non-substantiated/non-threshold report, and (4) no child protection contact. While there are important differences between non-substantiated and non-threshold reports, these were combined into one group because (a) non-threshold reports is a smaller category (n=3,998) with a low proportion of self-harm/suicidal ideation incidents meaning that some cell sizes would be too small to report, and (b) in previous studies of our cohort we have not found significant differences between children with non-substantiated and non-threshold reports in terms of mental health outcomes (e.g., O'Hare et al., 2021).

Age of first self-harm or suicidal ideation incident was estimated using information available for the month/year of birth and date of first recorded self-harm or suicidal ideation in any data collection. Cumulative incidence was calculated for each of the four levels of child protection contact as the cumulative number of individuals at each year interval with an incident of self-harm and/or suicidal ideation, divided by the total number of individuals in that child protection group and multiplied by 100 to give a percentage of each child protection group that had experienced self-harm and/or suicidal ideation in each age bracket.

References

O'Hare, K., Hussain, A., Laurens, K. R., et al. (2021). Self-reported mental health of children known to child protection services: an Australian population-based record linkage study. *Eur Child Adolesc Psychiatry* 2023; 32: 101-112.

Table 1. Cumulative frequencies and cumulative relative frequencies of suicidal ideation or self-harm (*N*=91,597)

Age (years)	No child protection contact	Non-substantiated/ non-threshold report	Substantiated report	Out-of-home care
Total number	**64,851**	**20,083**	**4,325**	**2,338**
**Self-harm**				
1	<15 (-)	<15 (-)	<15 (-)	<15 (-)
2	<15 (-)	<15 (-)	<15 (-)	<15 (-)
3	<15 (-)	<15 (-)	<15 (-)	<15 (-)
4	<15 (-)	<15 (-)	<15 (-)	<15 (-)
5	<15 (-)	<15 (-)	<15 (-)	<15 (-)
6	<15 (-)	<15 (-)	<15 (-)	<15 (-)
7	<15 (-)	<15 (-)	<15 (-)	<15 (-)
8	<15 (-)	<15 (-)	<15 (-)	<15 (-)
9	<15 (-)	<15 (-)	<15 (-)	<15 (-)
10	<15 (-)	<15 (-)	<15 (-)	<15 (-)
11	<15 (-)	<15 (-)	<15 (-)	<15 (-)
12	16 (<0.1%)	32 (0.2%)	16 (0.4%)	21 (0.9%)
13	36 (0.1%)	77 (0.4%)	54 (1.2%)	44 (1.9%)
14	59 (0.1%)	162 (0.8%)	94 (2.2%)	70 (3.0%)
15	111 (0.2%)	270 (1.3%)	148 (3.4%)	99 (4.2%)
16	191 (0.3%)	362 (1.8%)	193 (4.5%)	120 (5.1%)
**Suicidal ideation only***				
1	<15 (-)	<15 (-)	<15 (-)	<15 (-)
2	<15 (-)	<15 (-)	<15 (-)	<15 (-)
3	<15 (-)	<15 (-)	<15 (-)	<15 (-)
4	<15 (-)	<15 (-)	<15 (-)	<15 (-)
5	<15 (-)	<15 (-)	<15 (-)	<15 (-)
6	<15 (-)	<15 (-)	<15 (-)	<15 (-)
7	<15 (-)	<15 (-)	<15 (-)	<15 (-)
8	<15 (-)	<15 (-)	<15 (-)	<15 (-)
9	<15 (-)	<15 (-)	<15 (-)	<15 (-)
10	<15 (-)	<15 (-)	<15 (-)	<15 (-)
11	<15 (-)	<15 (-)	26 (0.1%)	<15 (-)
12	25 (<0.1%)	49 (0.2%)	35 (0.8%)	26 (1.1%)
13	64 (0.1%)	111 (0.6%)	64 (1.5%)	51 (2.2%)
14	130 (0.2%)	228 (1.1%)	124 (2.9%)	92 (4.0%)
15	226 (0.3%)	380 (1.9%)	201 (4.6%)	135 (5.8%)
16	371 (0.6%)	555 (2.8%)	257 (5.9%)	184 (7.8%)
Self-harm and/or suicidal ideation				
1	<15 (-)	<15 (-)	<15 (-)	<15 (-)
2	<15 (-)	<15 (-)	<15 (-)	<15 (-)
3	<15 (-)	<15 (-)	<15 (-)	<15 (-)
4	<15 (-)	<15 (-)	<15 (-)	<15 (-)
5	<15 (-)	<15 (-)	<15 (-)	<15 (-)
6	<15 (-)	<15 (-)	<15 (-)	<15 (-)
7	<15 (-)	<15 (-)	<15 (-)	<15 (-)
8	<15 (-)	<15 (-)	<15 (-)	<15 (-)
9	<15 (-)	<15 (-)	<15 (-)	<15 (-)
10	<15 (-)	20 (0.1%)	<15 (-)	<15 (-)
11	<15 (-)	41 (0.2%)	19 (0.4%)	20 (0.9%)
12	41 (0.1%)	90 (0.4%)	59 (1.4%)	52 (2.2%)
13	102 (0.2%)	205 (1.0%)	129 (3.0%)	100 (4.3%)
14	192 (0.3%)	409 (2.0%)	229 (5.3%)	171 (7.3%)
15	342 (0.5%)	666 (3.3%)	362 (8.4%)	239 (10.2%)
16	562 (0.9%)	917 (4.6%)	450 (10.4%)	304 (13.0%)

All numbers and percentages are cumulative. Cell sizes of <15 are suppressed. *Excludes individuals with both suicidal ideation and self-harm.

Figure. Cumulative Incidence of first self-harm or suicidal ideation event in (A) total sample, (B) girls only, and (C) boys only*

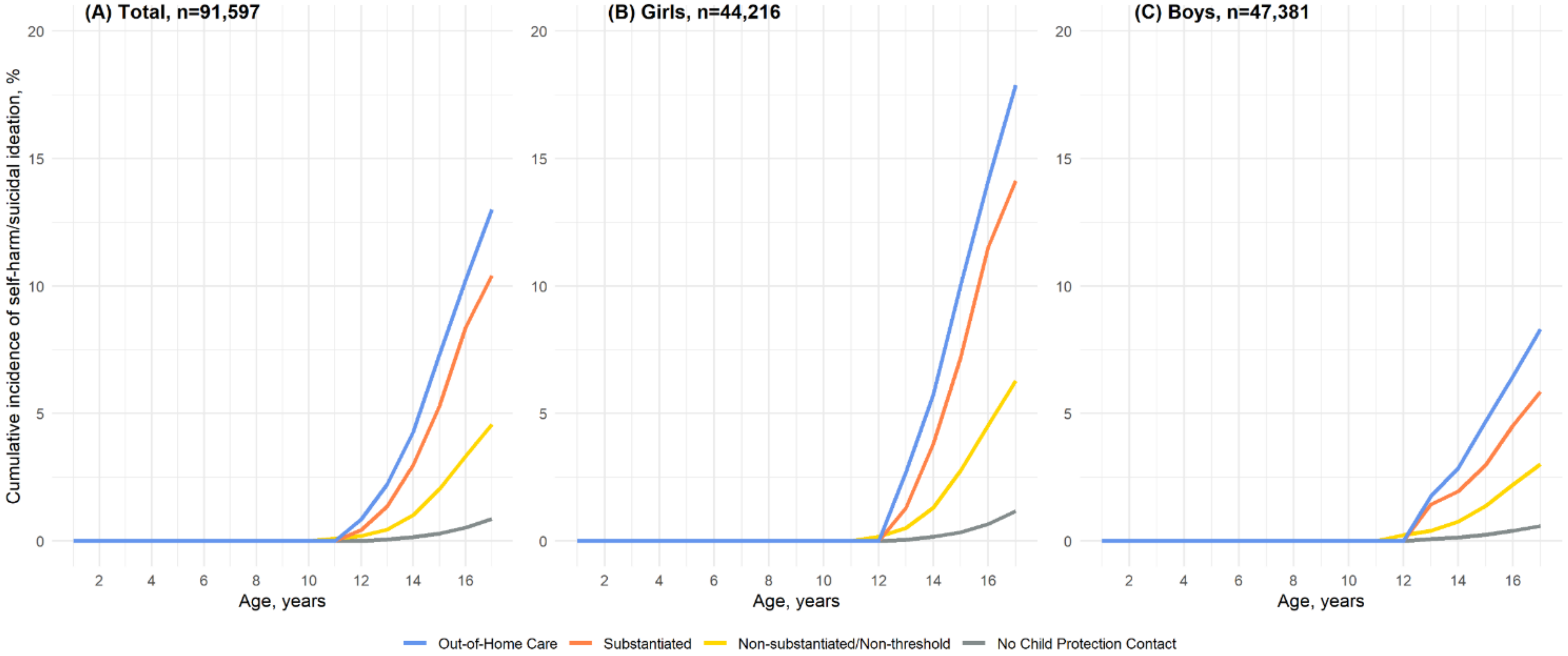

* The data on which these graphs are based are provided in table 2.

Table 2. Cumulative frequencies and cumulative relative frequencies of suicidal ideation and/or self-harm in girls and boys

	**Girls (*N*=44,216)**				**Boys (*N*=47,381)**			
**Age (years)**	**No child protection contact**	**Non-substantiated/ non-threshold report**	**Substantiated report**	**Out-of-home care**	**No child protection contact**	**Non-substantiated/ non-threshold report**	**Substantiated report**	**Out-of-home care**
Total number	**31,127**	**9,565**	**2,378**	**1,146**	**33,724**	**2,085**	**1,947**	**1,192**
1	<15 (-)	<15 (-)	<15 (-)	<15 (-)	<15 (-)	<15 (-)	<15 (-)	<15 (-)
2	<15 (-)	<15 (-)	<15 (-)	<15 (-)	<15 (-)	<15 (-)	<15 (-)	<15 (-)
3	<15 (-)	<15 (-)	<15 (-)	<15 (-)	<15 (-)	<15 (-)	<15 (-)	<15 (-)
4	<15 (-)	<15 (-)	<15 (-)	<15 (-)	<15 (-)	<15 (-)	<15 (-)	<15 (-)
5	<15 (-)	<15 (-)	<15 (-)	<15 (-)	<15 (-)	<15 (-)	<15 (-)	<15 (-)
6	<15 (-)	<15 (-)	<15 (-)	<15 (-)	<15 (-)	<15 (-)	<15 (-)	<15 (-)
7	<15 (-)	<15 (-)	<15 (-)	<15 (-)	<15 (-)	<15 (-)	<15 (-)	<15 (-)
8	<15 (-)	<15 (-)	<15 (-)	<15 (-)	<15 (-)	<15 (-)	<15 (-)	<15 (-)
9	<15 (-)	<15 (-)	<15 (-)	<15 (-)	<15 (-)	<15 (-)	<15 (-)	<15 (-)
10	<15 (-)	<15 (-)	<15 (-)	<15 (-)	<15 (-)	<15 (-)	<15 (-)	<15 (-)
11	<15 (-)	16 (0.2%)	<15 (-)	<15 (-)	<15 (-)	25 (0.2%)	<15 (-)	<15 (-)
12	15 (<0.1%)	48 (0.5%)	31 (1.3%)	31 (2.7%)	26 (0.1%)	42 (0.4%)	28 (1.4%)	21 (1.8%)
13	54 (0.2%)	126 (1.3%)	91 (3.8%)	66 (5.8%)	48 (0.1%)	79 (0.8%)	38 (2.0%)	34 (2.9%)
14	107 (0.3%)	265 (2.8%)	171 (7.2%)	115 (10.0%)	85 (0.3%)	144 (1.4%)	58 (3.0%)	56 (4.7%)
15	206 (0.7%)	434 (4.5%)	274 (11.5%)	162 (14.1%)	136 (0.4%)	232 (2.2%)	88 (4.5%)	77 (6.5%)
16	363 (1.2%)	601 (6.3%)	336 (14.1%)	205 (17.9%)	199 (0.6%)	316 (3.0%)	114 (5.9%)	99 (8.3%)

Cell sizes of <15 are suppressed.
